# Biliothorax in a patient with unresectable cholangiocarcinoma

**DOI:** 10.1002/rcr2.1239

**Published:** 2023-10-24

**Authors:** Sara Valero, Àlison Copoví, Ignacio Boira, Jose N. Sancho‐Chust, Violeta Esteban, Alejandro de la Paz, Eusebi Chiner

**Affiliations:** ^1^ Department of Medical Oncology Hospital Universitario de San Juan Alicante Spain; ^2^ Department of Respiratory Medicine Hospital Universitario de San Juan Alicante Spain

**Keywords:** biliary pleural effusion, Biliothorax, cholangiocarcinoma, percutaneous biliary drainage

## Abstract

Biliothorax is a rare but serious condition, as the presence of bile is damaging and can lead to empyema. Here, we report a case of a 51‐year‐old man recently diagnosed with unresectable cholangiocarcinoma, admitted to the hospital for malignant obstructive jaundice. After interventional management of biliary obstruction, the patient developed a significant right pleural effusion compatible with biliothorax, successfully managed with pleural drainage and antibiotic therapy. Resolution was possible with a conservative approach: biliary decompression, chest tube drainage and antibiotics.

## Graphical abstract

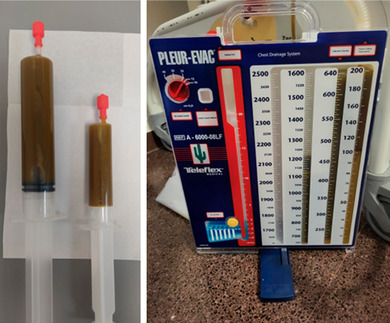

## INTRODUCTION

Biliothorax is a very rare entity characterized by the presence of bile in the pleural cavity, sometimes due to the existence of a biliopleural fistula (BPF).^1^ Among its causes is the complication of hepatobiliary procedures.^2^, ^3^ Two factors have been proposed for the cause of BPF.^2^, ^3^ The first one would be the accidental puncture of the pleural cavity during the percutaneous biliary drainage (PBD) placement. The second one would be related to the biliary tract obstruction, leading the bile to drain form a high‐pressure system to the pleural space.

We present the case of a patient with a history of unresectable locally advanced cholangiocarcinoma, complicated by the development of a biliothorax.

## CASE REPORT

**FIGURE 1:**
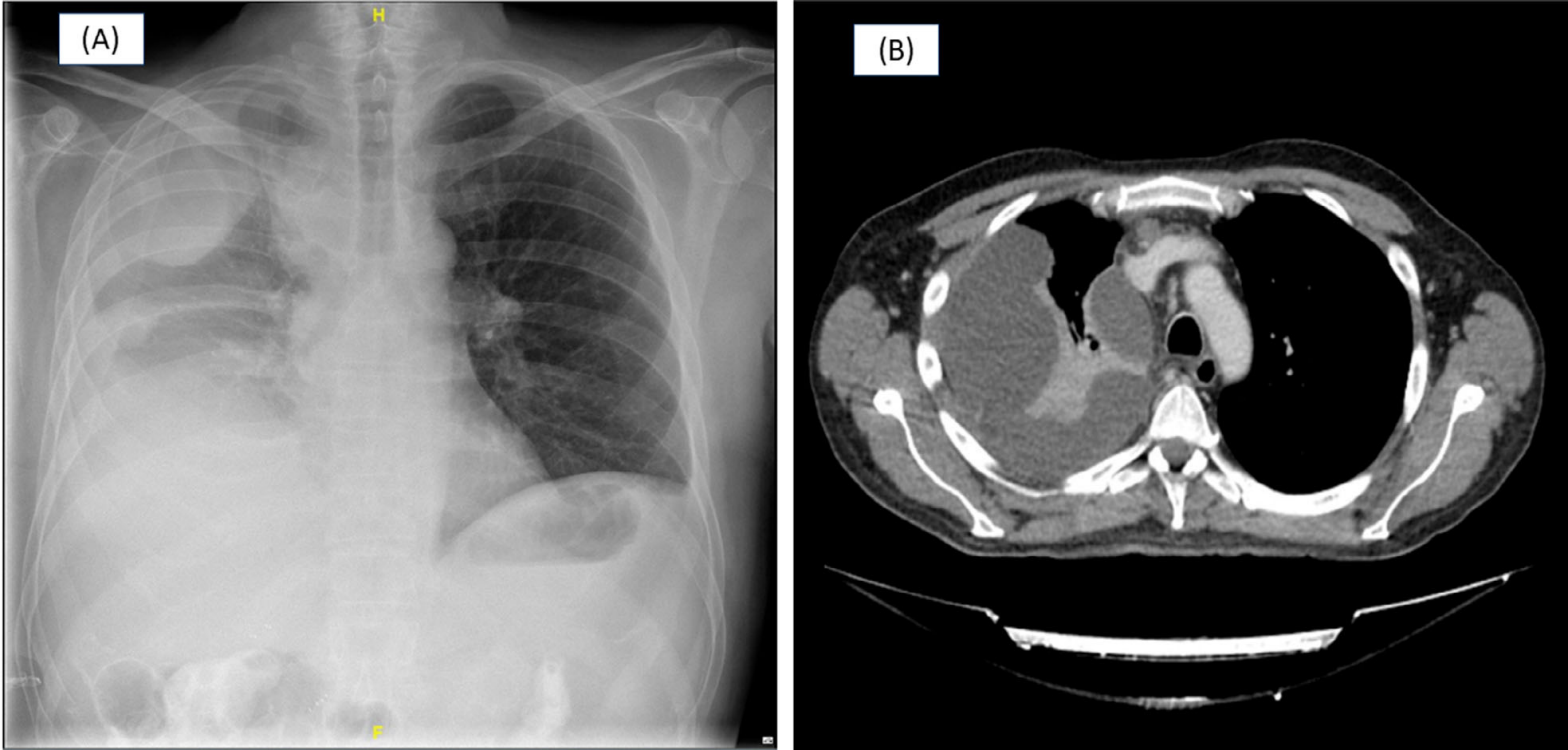
(A) Anteroposterior chest radiograph showing significant right loculated pleural effusion. (B) Computed axial tomography image of the chest showing significant right pleural effusion of loculated appearance occupying most of the right hemithorax except for a part of the pulmonary apex and middle lobe, with passive atelectasis of the adjacent lung parenchyma.

**FIGURE 2:**
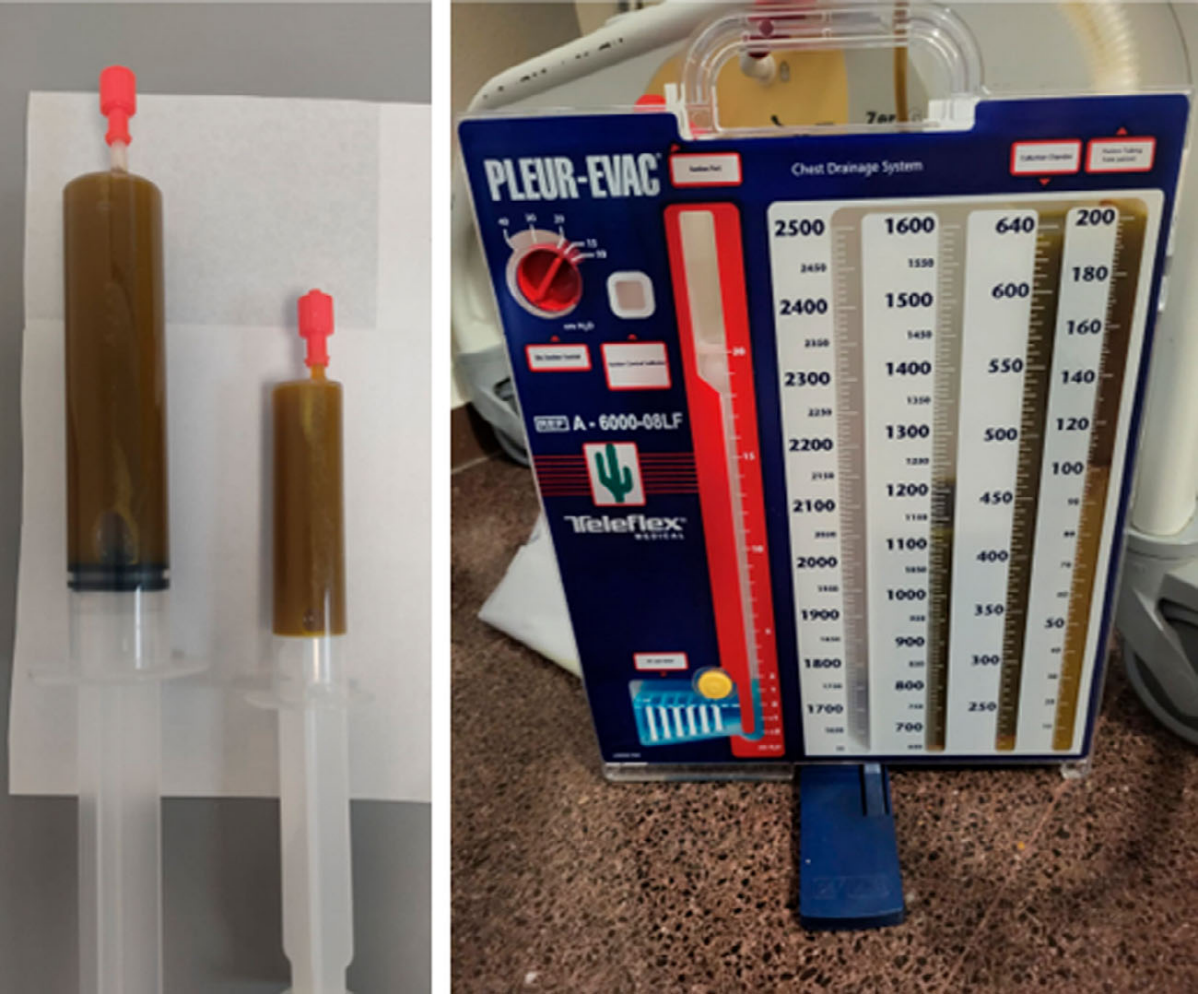
Bilious and purulent pleural fluid thoracentesis sample and in pleural drainage device.

A 51‐year‐old man diagnosed with locally advanced unresectable cholangiocarcinoma, was admitted to the hospital for malignant obstructive jaundice and a PBD was placed. After an initial favourable clinical course, the patient presented with abdominal pain, increased proportion of conjugated bilirubin in plasma and jaundice, and PBD occlusion was found. Biliary obstruction was managed with biliary dilatation, gastrojejunal bypass, replacement of the BPD for a biliary stent, and finally a metallic biliary stent. Four days after the last procedure, he presented clinical worsening with severe dyspnea and respiratory failure. On examination there were decreased breath sounds on right side of chest with dull percussion note. A chest X‐ray revealed a large and lobulated right pleural effusion (Figure 1A). The study was completed with a contrast thoracic computed tomography scan showing right pleural effusion and pulmonary infiltrates in the right lower lobe (Figure 1B). Diagnostic thoracentesis showed bilious and purulent pleural fluid (Figure 2), with a pH level of 6.6, total proteins of 1.8 g/dL, LDH of 15,224 U/L, leucocytes of 21,000/mm3 (90.6% of neutrophils), glucose of 9 mg/dL. Pleural total bilirubin of 19.48 mg/dL (plasma total bilirubin of 6.58 mg/dL) confirmed the suspicion of bilithorax. Pleural fluid cultures were positive for *Streptococcus anginosus* and *Klebsiella pneumoniae*. Pleural effusion was managed conservatively with pleural drainage and broad‐spectrum antibiotic therapy. Immediate tube thoracostomy was done and a total of 4000 mL of bilious and purulent pleural fluid was drained. Broad‐spectrum antibiotic treatment was started with meropenem 1 g/8 h and linezolid 600 mg/12 h, which was later adjusted to amoxicillin‐clavulanic acid based on antibiogram, receiving a total of 21 days of antibiotic treatment.

The patient had a favourable evolution, finally being discharged from the hospital. One month later, the patient was able to start chemotherapy treatment with cisplatin and gemcitabine from the department of medical oncology.

## DISCUSSION

Cholangiocarcinoma is the most common biliary tract malignancy and the second most common primary hepatic malignancy.^4^ They generally have a poor prognosis and most are diagnosed at an advanced stage of the disease. They can be classified as intrahepatic, perihilar or distal depending on where they arise in the biliary tree. In perihilar cholangiocarcinoma and distal cholangiocarcinoma, the most common presentation is jaundice due to obstruction of the biliary tree, usually at an earlier stage than in intrahepatic cholangiocarcinoma.

Biliothorax, defined as the presence of bile in the pleural fluid, is a rare cause of exudative pleural effusion. It was first reported in 1971 and since then about 70 cases have been described. Most of them are associated with malignant or benign biliary obstruction, pleurobiliary fistulas after hepatobiliary procedures (surgery or percutaneous drainage) and hepatic or subphrenic abscesses.^1^, ^2^, ^3^, ^5^ The right pleural cavity is usually involved due to anatomical proximity, but bile can also drain naturally through the oesophageal and aortic hiatuses, which can involve the left pleural cavity.^5^

The most specific diagnostic criteria is the ratio of total pleural fluid bilirubin to total serum bilirubin, which, when it is greater than 1, confirms the diagnosis of biliothorax. In a study of a total of 12 cases of biliothorax, the sensitivity of this diagnostic test was shown to be 76.9% when the result was greater than 1.^3^ In our case, the low level of proteins in the pleural fluid obtained is striking, we think that is secondary to cancer cachexia and to a systemic inflammatory response. These values in pleural fluid correlate with the decreased levels of albumin and total protein in the blood. Some authors suggest the presence of glycolic acid (the main component of bile acid) in the pleural fluid as an additional criteria.^3^ The study mentioned above showed that the combination of the two tests (presence of glycolic acid and a ratio of total bilirubin in the pleural fluid to total bilirubin in the serum greater than 1) gave a sensitivity of 100% in the diagnosis of biliothorax, although this result is limited because a positive glycolic acid test was obtained in only three patients of the study.

Bile acid is a potent chemo‐irritant and, its presence in the pleural space can cause a significant inflammatory response providing a favourable environment for infection leading to empyema.^1^ Hence it is imperative to recognize early and treat appropriately.

Biliothorax should be suspected in the presence of pleural effusion in patients who have undergone hepatobiliary procedures. An increase in the proportion of conjugated bilirubin in plasma with increasingly jaundice, can also point us to this condition.

The optimal treatment of biliothorax is operative, closing the biliopleural fistula with a high rate of success.^1^ In the last years, there is a trend towards a conservative management with chest tube drainage and biliary stenting to reduce ductal pressure and promote spontaneous BPF closure.^2^, ^3^

In conclusion, biliothorax is a rare complication of biliary obstruction that requires immediate attention, as biliothorax has a high propensity to be associated with empyema. Conservative non‐surgical treatment and adequate biliary drainage may be appropriate in these patients.

## AUTHOR CONTRIBUTIONS

Sara Valero, Àlison Copoví, Violeta Esteban and Ignacio Boira drafted the manuscript. Jose N. Sancho‐Chust, Alejandro De La Paz and Eusebi Chiner revised the manuscript. All authors approved the final manuscript.

## CONFLICT OF INTEREST STATEMENT

None declared.

## ETHICS STATEMENT

The authors declare that appropriate written informed consent was obtained for the publication of this manuscript and accompanying images.

## Data Availability

Research data are not shared.

## References

[1] Strange C , Allen ML , Freedland PN , Cunningham J , Sahn SA . Biliopleural fistula as a complication of percutaneous biliary drainage: experimental evidence for pleural inflammation. Am Rev Respir Dis. 1988;137(4):959–961. 10.1164/ajrccm/137.4.959 3355006

[2] Lee MT , Hsi SC , Hu P , Liu KY . Biliopleural fistula: a rare complication of percutaneous transhepatic gallbladder drainage. World J Gastroenterol. 2007;13(23):3268–3270. 10.3748/wjg.v13.i23.3268 17589912 PMC4436619

[3] Saraya T , Light RW , Sakuma S , Nakamoto Y , Wasa S , Ishida M , et al. A new diagnostic approach for bilious pleural effusion. Respir Investig. 2016;54(5):364–368. 10.1016/j.resinv.2016.03.009 27566385

[4] Razumilava N , Gores GJ . Cholangiocarcinoma. Lancet. 2014;383(9935):2168–2179. 10.1016/S0140-6736(13)61903-0 24581682 PMC4069226

[5] Briones‐Gómez A , Sánchez‐Samblancat M , Bakki A . A rare presentation of bilothorax. Arch Bronconeumol. 2023;59(7):447–448. 10.1016/j.arbres.2023.03.023 37069012

